# Automated in-silico detection of cell populations in flow cytometry readouts and its application to leukemia disease monitoring

**DOI:** 10.1186/1471-2105-7-282

**Published:** 2006-06-05

**Authors:** Joern Toedling, Peter Rhein, Richard Ratei, Leonid Karawajew, Rainer Spang

**Affiliations:** 1 Max Planck Institute for Molecular Genetics & Berlin Center for Genome Based Bioinformatics, Ihnestrasse. 73, D-14195 Berlin, Germany; 2 Dept. of Hematology, Oncology and Tumor Immunology, Robert-Roessle-Clinic at the HELIOS Klinikum Berlin, Charité Medical School, Berlin, Germany; 3 EMBL – European Bioinformatics Institute, Wellcome Trust Genome Campus, Hinxton, Cambridge CB10 1SD, UK

## Abstract

**Background:**

Identification of minor cell populations, e.g. leukemic blasts within blood samples, has become increasingly important in therapeutic disease monitoring. Modern flow cytometers enable researchers to reliably measure six and more variables, describing cellular size, granularity and expression of cell-surface and intracellular proteins, for thousands of cells per second. Currently, analysis of cytometry readouts relies on visual inspection and manual gating of one- or two-dimensional projections of the data. This procedure, however, is labor-intensive and misses potential characteristic patterns in higher dimensions.

**Results:**

Leukemic samples from patients with acute lymphoblastic leukemia at initial diagnosis and during induction therapy have been investigated by 4-color flow cytometry. We have utilized multivariate classification techniques, *Support Vector Machines *(SVM), to automate leukemic cell detection in cytometry. Classifiers were built on conventionally diagnosed training data. We assessed the detection accuracy on independent test data and analyzed marker expression of incongruently classified cells. SVM classification can recover manually gated leukemic cells with 99.78% sensitivity and 98.87% specificity.

**Conclusion:**

Multivariate classification techniques allow for automating cell population detection in cytometry readouts for diagnostic purposes. They potentially reduce time, costs and arbitrariness associated with these procedures. Due to their multivariate classification rules, they also allow for the reliable detection of small cell populations.

## Background

Flow cytometry has evolved to an indispensable tool in biology and medicine, with a significant impact on hematology. To date, diagnosis and classification of acute lymphocytic leukemia (ALL), depend on the flow-cytometric description of the leukemic cell clone. Recently, flow cytometry has also become an attractive approach for evaluation of therapy response and especially detection of minimal residual disease (MRD) [1]. Flow cytometry provides a quantitative cell description by a number of variables, including cell size, granularity and expression of cell-surface and intracellular proteins. Due to the continuous development of flow cytometric techniques, their readouts have become increasingly complex and require adequate analysis methods.

Current diagnostic evaluation of flow cytometry readouts relies on simplistic two-dimensional analysis techniques. The basis is a labor-intensive *gating *procedure. In a series of two-dimensional dot plots, leukemic cells are manually flagged by drawing polygons around regions, which are known to contain mostly leukemic cells. A large number of two-dimensional plots need to be inspected and several regions need to be defined manually. Finally, candidates for leukemic cells are those inside a Boolean combination of drawn regions, called *gate *[2,3]. From the data analysis perspective, gating lymphoblastic cells is a problem of supervised statistical learning. One starts with a training set of flow cytometry readouts, which are already gated by an expert. The challenge is to derive a multivariate classification model from this data, which is able to produce accurate gatings on different readouts that have not been pre-gated by an expert. The objects of classification are single cells that can either be leukemic or physiological lymphocytes. Typically, each cell is described by a 4–9 dimensional vector of flow cytometry measurements. With only a small number of pre-gated readouts, one already has several thousand training points. Problems are posed by the non-linear shape of the regions containing leukemic lymphocytes and the patient-to-patient variability of these regions.

To our knowledge, replacing the manual gating process by a computer-based automated multivariate analysis has not been described previously. Some cytometer software suites contain tools for automated *walk-away *analyses once the gates have been defined. These tools, however, are also restricted to two-dimensional decision rules. In addition, some methods to make use of cytometry readouts in a multivariate setting have been proposed. Here, we briefly review three of these methods and explain the differences to our concept.

Valet et al. [4] introduced a classification method for blood samples in flow cytometry, called *algorithmic data sieving*. For each class of samples, a discretized representative is derived from training data. New samples are then classified according to their similarity to these representatives. De Zen et al. [5] investigated the feasibility to classify acute-leukemia subtypes on flow-cytometry readouts. First, they determined the leukemic cells by a conventional, manual gating procedure and discarded all other cells from the data. For each sample, they summarized the measurements over all leukemic cells for each variable and used these summary values for classifying samples with linear discriminant analysis [6]. Roederer and Hardy [7] proposed an algorithm for sample comparison based on cytometry readouts. Their algorithm identifies multi-dimensional hyper-rectangular bins that significantly differ in the proportion of cells contained between a test sample and a control sample. The union of all these regions comprises a *frequency difference gate*. This gate may be used to assign new samples to test or control group, as well as to find differences between similar types of cells under different conditions. While their approach could be modified to search for regions in multivariate space, which differ between leukemic and non-leukemic samples, they restrict these regions to be rectangles, which is not the case in conventional gating [3]. Moreover, in this case regions are not required to contain the same proportion of cells but rather to contain mostly cells of the same class. In contrast to these approaches, we are not interested in classifying blood samples based on their cytometry readouts, but rather in automated identification of cell populations within the samples. We report on the applicability of statistical learning methodology, for achieving automated, reliable in-silico gatings on flow cytometry readouts. To this aim, we employ supervised classification with *Support Vector Machines *[8].

## Results

### Algorithm

For supervised classification of the leukemic status of cells, we employ a Support Vector Machine based algorithm that allows for non-linear decision boundaries in the input space spanned by the cells' measured characteristics and protein expression levels. Our algorithm takes into account outstanding properties of flow-cytometry readout data, namely

• samples consisting of tens of thousands of individual observations

• large inter-sample variation due to non-standardized methods of obtaining measurements.

#### Support Vector Machines

*Support Vector Machines *(SVM) [9,10] are a class of regularized multivariate classification models that are widely used for predictive modelling of multidimensional data. We provide a quick review of SVM here. Let ***X ***be the data matrix holding *n *observations ***x***_*i*_, with *i *∈ 1,..., *n*, in columns and *p *variables in rows. The observations ***x***_*i *_are said to reside in a *p*-dimensional input space. For each observation ***x***_*i *_its class (clinical phenotype) *y*_*i *_∈ {± 1} is known beforehand in case of the training set or to be predicted in case of the test set. SVM fit a maximal (soft) margin hyperplane between the two classes. With high-dimensional problems, there may be several perfectly separating hyperplanes (the maximum likelihood approach leads to an ill-posed problem). There is, however, only one separating hyperplane with maximal distance to the nearest training points of either class.

More formally, among all hyperplanes of the form

**w**·***x ***+ *b *= 0 | **w **∈ ℝ^*N*^, *b *∈ ℝ

corresponding to linear decision functions

*c*(***x***_*j*_) = sgn(**w**·***x***_*j *_+ *b*)

there exists one that maximizes the distance of each input vector to the hyperplane. It can be shown, that this *optimal hyperplane *can be empirically obtained from data ***X ***by solving

min⁡w,b{12‖w‖2}subject toyi⋅(w⋅xi+b)≥1, i=1,…,n
 MathType@MTEF@5@5@+=feaafiart1ev1aaatCvAUfKttLearuWrP9MDH5MBPbIqV92AaeXatLxBI9gBaebbnrfifHhDYfgasaacH8akY=wiFfYdH8Gipec8Eeeu0xXdbba9frFj0=OqFfea0dXdd9vqai=hGuQ8kuc9pgc9s8qqaq=dirpe0xb9q8qiLsFr0=vr0=vr0dc8meaabaqaciaacaGaaeqabaqabeGadaaakeaafaqabeqadaaabaWaaCbeaeaacyGGTbqBcqGGPbqAcqGGUbGBaSqaaGqabiab=Dha3jabcYcaSiabdkgaIbqabaGcdaGadaqaamaalaaabaGaeGymaedabaGaeGOmaidaamaafmaabaGae83DaChacaGLjWUaayPcSdWaaWbaaSqabeaacqaIYaGmaaaakiaawUhacaGL9baaaeaacqqGZbWCcqqG1bqDcqqGIbGycqqGQbGAcqqGLbqzcqqGJbWycqqG0baDcqqGGaaicqqG0baDcqqGVbWBaeaacqWG5bqEdaWgaaWcbaGaemyAaKgabeaakiabgwSixlabcIcaOiab=Dha3jabgwSixJqadiab+Hha4naaBaaaleaacqWGPbqAaeqaaOGaey4kaSIaemOyaiMaeiykaKIaeyyzImRaeGymaeJaeiilaWIaeeiiaaIaemyAaKMaeyypa0JaeGymaeJaeiilaWIaeSOjGSKaeiilaWIaemOBa4gaaaaa@67D6@

where

w=∑i=1nαiyixi|αi∈ℝ.
 MathType@MTEF@5@5@+=feaafiart1ev1aaatCvAUfKttLearuWrP9MDH5MBPbIqV92AaeXatLxBI9gBaebbnrfifHhDYfgasaacH8akY=wiFfYdH8Gipec8Eeeu0xXdbba9frFj0=OqFfea0dXdd9vqai=hGuQ8kuc9pgc9s8qqaq=dirpe0xb9q8qiLsFr0=vr0=vr0dc8meaabaqaciaacaGaaeqabaqabeGadaaakeaaieqacqWF3bWDcqGH9aqpdaaeWbqaaGGaciab+f7aHnaaBaaaleaacqWGPbqAaeqaaOGaemyEaK3aaSbaaSqaaiabdMgaPbqabaacbmGccqqF4baEdaWgaaWcbaGaemyAaKgabeaaaeaacqWGPbqAcqGH9aqpcqaIXaqmaeaacqWGUbGBa0GaeyyeIuoakiabcYha8jab+f7aHnaaBaaaleaacqWGPbqAaeqaaOGaeyicI48efv3ySLgznfgDOjdaryqr1ngBPrginfgDObcv39gaiqaacqaFDeIuieaacqWEUaGlaaa@5136@

In practice, *α*_*i *_will often be zero for many *i *and different from zero for only a limited number of *m *observations with *m *≤ *n*. These *m *observations solely define the separating hyperplane and are called *support vectors*. The maximal-margin concept is typically combined with the *kernel trick *to allow for flexible non-linear classification boundaries. The kernel trick is applicable to classification algorithms that can be expressed in terms of inner products of the inputs, as it is the case for the maximum-margin hyperplane. The inner products are substituted by a *kernel function k*(***x***_*i*_, ***x***_*j*_), which corresponds to a *feature map *Φ that maps the profiles from the input space into a *feature space *ℋ
 MathType@MTEF@5@5@+=feaafiart1ev1aaatCvAUfKttLearuWrP9MDH5MBPbIqV92AaeXatLxBI9gBamrtHrhAL1wy0L2yHvtyaeHbnfgDOvwBHrxAJfwnaebbnrfifHhDYfgasaacH8akY=wiFfYdH8Gipec8Eeeu0xXdbba9frFj0=OqFfea0dXdd9vqai=hGuQ8kuc9pgc9s8qqaq=dirpe0xb9q8qiLsFr0=vr0=vr0dc8meaabaqaciaacaGaaeqabaWaaeGaeaaakeaaimaacqWFlecsaaa@3763@:

Φ: ℝ^*p *^→ ℋ
 MathType@MTEF@5@5@+=feaafiart1ev1aaatCvAUfKttLearuWrP9MDH5MBPbIqV92AaeXatLxBI9gBamrtHrhAL1wy0L2yHvtyaeHbnfgDOvwBHrxAJfwnaebbnrfifHhDYfgasaacH8akY=wiFfYdH8Gipec8Eeeu0xXdbba9frFj0=OqFfea0dXdd9vqai=hGuQ8kuc9pgc9s8qqaq=dirpe0xb9q8qiLsFr0=vr0=vr0dc8meaabaqaciaacaGaaeqabaWaaeGaeaaakeaaimaacqWFlecsaaa@3763@

***x ***↦ Φ(*x*).

This results in the original algorithm being carried out in ℋ
 MathType@MTEF@5@5@+=feaafiart1ev1aaatCvAUfKttLearuWrP9MDH5MBPbIqV92AaeXatLxBI9gBamrtHrhAL1wy0L2yHvtyaeHbnfgDOvwBHrxAJfwnaebbnrfifHhDYfgasaacH8akY=wiFfYdH8Gipec8Eeeu0xXdbba9frFj0=OqFfea0dXdd9vqai=hGuQ8kuc9pgc9s8qqaq=dirpe0xb9q8qiLsFr0=vr0=vr0dc8meaabaqaciaacaGaaeqabaWaaeGaeaaakeaaimaacqWFlecsaaa@3763@ now and leads to non-linear decision boundaries in the input space. After application of the kernel trick, the decision functions are of the more general form

c(xj)=sgn⁡(∑inyiαik(xj,xi)+b).
 MathType@MTEF@5@5@+=feaafiart1ev1aaatCvAUfKttLearuWrP9MDH5MBPbIqV92AaeXatLxBI9gBaebbnrfifHhDYfgasaacH8akY=wiFfYdH8Gipec8Eeeu0xXdbba9frFj0=OqFfea0dXdd9vqai=hGuQ8kuc9pgc9s8qqaq=dirpe0xb9q8qiLsFr0=vr0=vr0dc8meaabaqaciaacaGaaeqabaqabeGadaaakeaacqWGJbWycqGGOaakieWacqWF4baEdaWgaaWcbaGaemOAaOgabeaakiabcMcaPiabg2da9iGbcohaZjabcEgaNjabc6gaUnaabmaabaWaaabCaeaacqWG5bqEdaWgaaWcbaGaemyAaKgabeaaiiGakiab+f7aHnaaBaaaleaacqWGPbqAaeqaaOGaem4AaSMaeiikaGIae8hEaG3aaSbaaSqaaiabdQgaQbqabaGccqGGSaalcqWF4baEdaWgaaWcbaGaemyAaKgabeaakiabcMcaPiabgUcaRiabdkgaIbWcbaGaemyAaKgabaGaemOBa4ganiabggHiLdaakiaawIcacaGLPaaacqGGUaGlaaa@51DA@

#### Soft margin classification

Noise may cause large overlap of the classes even in the feature space, such that a perfectly separating hyperplane may not exist. In this case, one can allow for misclassifications (margin violations) by relaxing the optimization constraints to

*y*_*i*_·(**w**·*x*_*i *_+ *b*) ≥ 1 - *ξ*_*i*_, *i *= 1,..., *n*.

*ξ*_*i *_≥ 0 are commonly called *slack variables*. The optimal *soft *margin classifier is then found by minimizing the regularized risk function

r(w,ξ)=12‖w‖2+C∑i=1nξi.
 MathType@MTEF@5@5@+=feaafiart1ev1aaatCvAUfKttLearuWrP9MDH5MBPbIqV92AaeXatLxBI9gBaebbnrfifHhDYfgasaacH8akY=wiFfYdH8Gipec8Eeeu0xXdbba9frFj0=OqFfea0dXdd9vqai=hGuQ8kuc9pgc9s8qqaq=dirpe0xb9q8qiLsFr0=vr0=vr0dc8meaabaqaciaacaGaaeqabaqabeGadaaakeaacqWGYbGCcqGGOaakieqacqWF3bWDcqGGSaaliiGacqGF+oaEcqGGPaqkcqGH9aqpdaWcaaqaaiabigdaXaqaaiabikdaYaaadaqbdaqaaiab=Dha3bGaayzcSlaawQa7amaaCaaaleqabaGaeGOmaidaaOGaey4kaSIaem4qam0aaabCaeaacqGF+oaEdaWgaaWcbaGaemyAaKgabeaaaeaacqWGPbqAcqGH9aqpcqaIXaqmaeaacqWGUbGBa0GaeyyeIuoakiabc6caUaaa@49BC@

In this formulation, perfect separability of the two classes is not required and margin violations are allowed. The trade off between margin violations and margin size is reflected by the regularization (cost) parameter *C*.

Regularization is essential to counter the additional flexibility acquired by use of the kernel trick.

In recent years, SVM have proven to be a powerful and robust classification method that can handle various kinds of input data [11]. For a more extensive introduction to SVM see, e.g, [8].

We chose a radial-basis kernel function, which can be defined as:

*k*_*γ*_(***x***_*i*_, ***x***_*j*_) = exp(-*γ*·||***x***_*i *_- ***x***_*j*_||^2^)

where *x*_*i *_and ***x***_*j *_are two input data points and *γ *is the inverse band width of the smoothing kernel.

#### Building the classifier

With our data, we applied the SVM to separate leukemic from non-leukemic cells. The optimal settings for the SVM parameters *C *and *γ *were determined on set-aside calibration data, while the actual performance of the SVM classifier was analyzed on another set-aside test data set. Since our samples each contained up to 300,000 cells, requiring highly demanding computations, we had to think about methods of data reduction. Since with SVM classification, the decision boundary is only determined by the *support vectors *[[8], chap. 1], we can discard all observations that are not support vectors from the training data. We split the data into subsets, keeping only the support vectors from each subset and build the final SVM classifier on the sets of support vectors, similar to Boser et al. [12]. In detail, we used the following procedure to estimate the test error of the learned classifier:

1. Split data into 50% training set, 25% calibration set, and 25% test set.

2. For a reasonable number of possible parameter settings Θ_*k *_= (*γ*_*k*_, *C*_*k*_):

(a) initialize an empty set of training vectors T
 MathType@MTEF@5@5@+=feaafiart1ev1aaatCvAUfKttLearuWrP9MDH5MBPbIqV92AaeXatLxBI9gBamrtHrhAL1wy0L2yHvtyaeHbnfgDOvwBHrxAJfwnaebbnrfifHhDYfgasaacH8akY=wiFfYdH8Gipec8Eeeu0xXdbba9frFj0=OqFfea0dXdd9vqai=hGuQ8kuc9pgc9s8qqaq=dirpe0xb9q8qiLsFr0=vr0=vr0dc8meaabaqaciaacaGaaeqabaWaaeGaeaaakeaaimaacqWFtepvaaa@3847@_*k *_= { }

(b) split training set into computationally feasible subsets

(c) for each of these subsets

• learn SVM-classifier for leukemic versus non-leukemic cells

• identify support vectors *S *on this subset

• include these support vectors into the set of training vectors T
 MathType@MTEF@5@5@+=feaafiart1ev1aaatCvAUfKttLearuWrP9MDH5MBPbIqV92AaeXatLxBI9gBamrtHrhAL1wy0L2yHvtyaeHbnfgDOvwBHrxAJfwnaebbnrfifHhDYfgasaacH8akY=wiFfYdH8Gipec8Eeeu0xXdbba9frFj0=OqFfea0dXdd9vqai=hGuQ8kuc9pgc9s8qqaq=dirpe0xb9q8qiLsFr0=vr0=vr0dc8meaabaqaciaacaGaaeqabaWaaeGaeaaakeaaimaacqWFtepvaaa@3847@_*k *_= T
 MathType@MTEF@5@5@+=feaafiart1ev1aaatCvAUfKttLearuWrP9MDH5MBPbIqV92AaeXatLxBI9gBamrtHrhAL1wy0L2yHvtyaeHbnfgDOvwBHrxAJfwnaebbnrfifHhDYfgasaacH8akY=wiFfYdH8Gipec8Eeeu0xXdbba9frFj0=OqFfea0dXdd9vqai=hGuQ8kuc9pgc9s8qqaq=dirpe0xb9q8qiLsFr0=vr0=vr0dc8meaabaqaciaacaGaaeqabaWaaeGaeaaakeaaimaacqWFtepvaaa@3847@_*k *_∪ *S*

(d) learn SVM-classifier on T
 MathType@MTEF@5@5@+=feaafiart1ev1aaatCvAUfKttLearuWrP9MDH5MBPbIqV92AaeXatLxBI9gBamrtHrhAL1wy0L2yHvtyaeHbnfgDOvwBHrxAJfwnaebbnrfifHhDYfgasaacH8akY=wiFfYdH8Gipec8Eeeu0xXdbba9frFj0=OqFfea0dXdd9vqai=hGuQ8kuc9pgc9s8qqaq=dirpe0xb9q8qiLsFr0=vr0=vr0dc8meaabaqaciaacaGaaeqabaWaaeGaeaaakeaaimaacqWFtepvaaa@3847@_*k*_

(e) use learned classifier on calibration set to compute the *calibration error ϕ*_*k*_

3. Keep learned classifier with lowest calibration error.

4. Use this classifier to predict test data.

5. Return prediction error on test set as *test error*.

Keeping only the support vectors from each data subset reduces the amounts of involved data and renders the computations feasible on common present-day computers.

The final test error provides an unbiased estimate of the classifier's prediction error (generalization error) on new data [13, chap. 7].

#### Artificial noise

We are interested in building classifiers that are insensitive to minor noise induced into experimental measurements by the experimental setup or measuring device. By generating artificial noise and applying it to training data, one tests the ability to learn a "correct" concept in the presence of noise [14]. Here, we add artificial noise to our training data to simulate such minor experimental variations. As noise, we take random normally distributed numbers. Classification rules that are built on noisy versions of the training data and apply to artificial-noise-free test data as well, have the potential to generalize better to new test samples [15].

### Application on patient data

In each of 37 patient samples (Table 1), the leukemic cells were identified by manual gating beforehand. We then randomly assigned 19 patient samples to a training set, while the remaining 18 patient samples made up the test set. We also included two other samples, for which the proportion of leukemic cells was predefined, into the training set. One of these samples originated from a non-leukemic blood sample (sample 38 in Table 1) while the other one was enriched with leukemic cells by Ficoll gradient-density centrifugation and subsequent manual filtering of the flow cytometry data upon visual inspection (sample 39 in Table 1).

From each sample of the training set, we randomly drew 10,000 cells and discarded all other cells. Thus, our training data consisted of 210,000 cells and the associated labels, either "leukemic" or "non-leukemic".

To simulate minor experiment-induced variations, we added random noise to the data. For each variable, we determined its standard deviation across all cells of the training data. We then drew random numbers from a normal distribution with mean 0 and standard deviation equal to 10% of the variable's observed standard deviation. These random numbers were added to the values of the training data.

On the noisy training data, we learned the optimal SVM classifier and evaluated its classification performance on the test set as well as on the two samples with predefined leukemic proportion.

For building the SVM classifier, we again split the training data into an actual training set, a calibration set and a test set to select the optimal parameter settings and to avoid overfitting.

**Figure 1:**
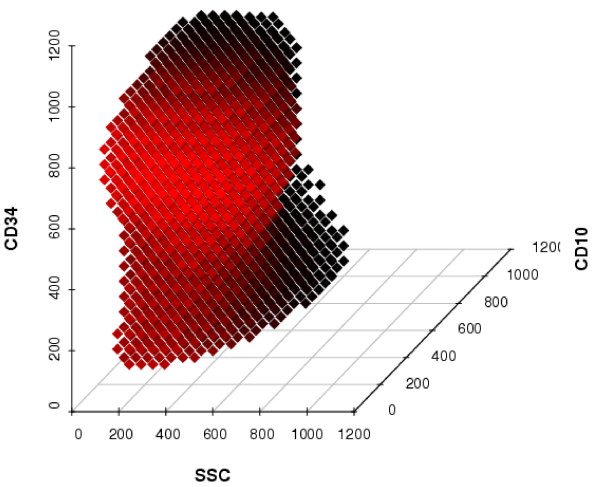
**SVM classification on a three-dimensional subspace**. Shown are points, at which cells would be classified as being leukemic by the learned SVM, in a three-dimensional subspace of the six-dimensional space spanned by all variables. The three variables not shown are fixed at their median value. The color of the points is meant to emphasize the three-dimensionality of the plot, with brighter points being closer to a hypothetical observer in front of the plot.

The SVM defines a region in six-dimensional space containing the leukemic cells. Due to the kernel trick, the classification boundary between points in the six-dimensional space, at which cells would be classified as being leukemic, and the other points, at which cells would be classified as physiological blood cells, is a non-linear structure. To illustrate this, we presenl a projection of this region on the three-dimensional subspace spanned by the variables SSC, CD34 and CD10 (Figure 1).

The SVM classifier employs a radial-basis kernel function with parameters *σ *= 0.5 and *C *= 4. However, changing each parameter selling by up to 25% had no effect on the classification performance, underlining the robustness of the method. The SVM classifier is based 958 support vectors, of which 478 are leukemic cells. On the training data, the learned radial-basis SVM classifier achieves a classification accuracy of 99.6%.

For comparison, we also evaluated the classification performance of an SVM classifier utilizing a linear kernel function instead of a radial basis one. We observed a slightly worse performance of this classifier on the training data, namely an error of 0.7%.

We used the SVM classifier to predict leukemic cells in the two samples, either without or enriched with leukemic cells, respectively. Cells from both samples had been used for learning the SVM as well, but we made sure that the same cells would not be used for testing the SVM performance. From the non-leukemic sample, we drew a subsample of 20,000 cells at random to avoid exceeding available RAM during computations for the SVM prediction. Of these 20,000 cells, only 2 (0.01%) were misclassified as being leukemic (sample 38, Table 1). From the enriched leukemic sample (containing approx. 94% leukemic blasts), we also drew a random subsample of 20,000 cells. Of these, the SVM classified 576 (2.88%) as being non-leukemic (sample 39, Table 1).

We utilized the learned SVM to predict the leukemic status of the cells in the test set, which consisted of 18 patient samples that had not been involved in training the classifier. We compared the predicted leukemia status with the one determined by gating beforehand. On this independent test set, the SVM achieved a *sensitivity *of 99.78% and a *specificity *of 98.87% for predicting the leukemic status of cells. In summary, for 0.94% of the cells of the test data, the leukemia status differed between SVM prediction and gating assignment. The total number of true and wrong predictions can be seen in Table 2.

To evaluate the robustness of the observed classification performance, we repeated the random splitting of patient samples into training and test set 500 times, and reran the full analysis for each split. The mean sensitivity was 98.06% (95% confidence interval [CI] 88.7% to 99.8%) and the mean specificity was 99.27% (95% CI 98.6% to 99.9%), which confirms the stability of our results. Across the 500 random splittings on average, we observed 2006 support vectors (sd: 496), which is more than for the original splitting.

**Figure 2:**
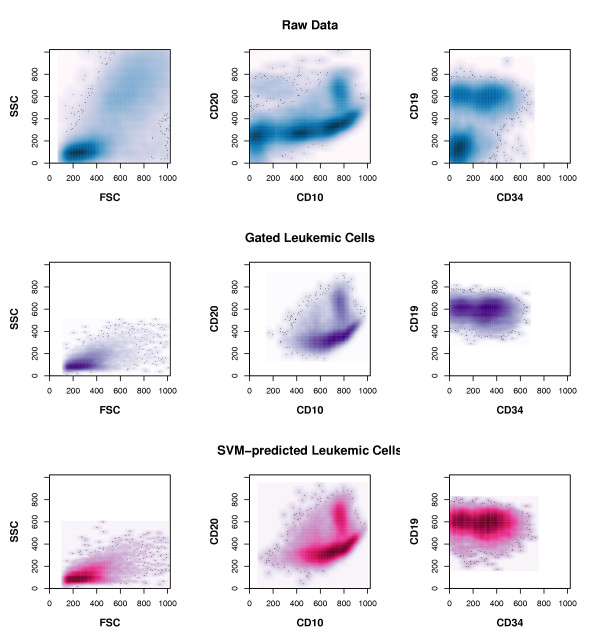
**Comparison of detected leukemic cells**. This figure displays the distribution of cells drawn at random from the test data that consists of 18 combined patient samples The plots in each row show the density distribution of the same cells with respect to their expression of the variables denoted on the axes. The darker an area in each plot, the more cells lie within that area. **Upper row**: All measured cells are shown. **Middle row**: Only cells deemed leukemic by manual gating are shown. **Lower row**: Only cells deemed leukemic by the built SVM classifier are shown.

To further assess the built classifier's precision, we took another independent peripheral blood sample, which was taken on the initial day of treatment, and separately measured aliquots from this sample with intervals of several minutes in between. Cytometer settings were not changed in between measurements. On each of the six readouts, we applied the classifier to predict the percentage of leukemic cells included. The predicted values ranged from 21.19% to 21.99% percent, similar to the manual-gating assigned percentage range that extented from 20.55% to 21.94%. We compared the spatial distribution of cells being classified as leukemic by the SVM to that of cells deemed leukemic due to manual gating and to the spatial distribution of all cells in the test data (see Figure 2). The distributions of cells deemed leukemic by manual gating and by SVM classification are nearly identical. However, the area in multivariate space, in which the SVM would assign cells to the leukemic class, is slightly larger than that defined by manual gating.

**Figure 3:**
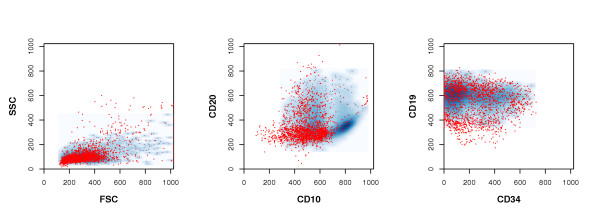
**Leukemic cells detected solely by the SVM**. In this figure, the distribution of those cells, which were deemed to be physiological blood cells by conventional gating, but classified as leukemic by the SVM (red dots) is displayed. For comparison, to the density distribution of those cells deemed leukemic by both methods is also shown (blue background densities). The darker a blue area is, the more confirmed leukemic cells lie within that area.

The large majority (96.8%) of the incongruently classified cells are deemed to be physiological blood cells by manual gating but predicted as being leukemic by the SVM. Most of these stem from samples taken before initial treatment (see Table 2). Their scatter and fluorescence measurements, compared to those cells deemed leukemic by both methods, can be seen in Figure 3.

## Discussion

Modern flow cytometers enable researchers to reliably measure six and more variables, such as size, shape and expression of cell-surface and intracellular proteins, for thousands of cells per second. In leukemia research, one is interested in the identification of leukemic cells, which are characterized by abnormal patterns of surface marker expression. The physiological co-expression patterns of these proteins during blood-cell development hints to a tight regulation of the expression of these markers. Searching for abnormal expression patterns with analysis techniques employing at most two markers at the same time has been successfully established in clinical leukemia research [3]. However, these techniques, such as gating, are labor-intensive, subjective and not able to capture higher-order dependencies between measured variables.

Some existing methods make use of the multivariate setting of cytometry readouts [4,5,7], but these methods aim at sample classification based on the readouts rather than on cell population detection within samples.

Here, we have shown the potential of well-established multivariate-analysis techniques, such as classification via SVM, to automate detection of leukemic cells in flow cytometry readouts from patients' bone marrow and peripheral blood samples. The SVM operates in the space spanned by all variables and even augments it by the use of the kernel trick [8]. Classification in this complex space takes into account higher-order dependencies between the variables, which are disregarded when restricting oneself to one- or two-dimensional decision rules. With flow cytometry, there is no denying that dependencies between the measured variables do exist, due to properties of utilized materials and biological, superordinate regulatory mechanisms. Developing blood cells are characterized by combinations of interacting surface markers [16]. Also, measured fluorescence intensities cannot be considered independent from each other because of overlaps between the fluorochromes' emission spectra [17]. Most cytometers can be set to compensate for these overlaps. While this compensation removes part of the influence of each measured variable on the others, one cannot expect it to remove every dependence between them.

We built a SVM classifier on the training data, containing approx. 50% of the available data. We, again, split the training data into separate sets for building the classifier, selecting the optimal parameter settings, and assessing the training error. This procedure and the artificial noise added to the training data prevent overfitting of the learned SVM classifier. The learned SVM had a very low training error of 0.4%. Remarkably, this error did not increase, when modifying the SVM's parameter sellings by up to 25%, indicating a very clear separation of leukemic and non-leukemic cells in the enhanced feature space.

We tested the learned radial-basis SVM classifier on independent test data, generated from a non-leukemic blood sample, an enriched leukemic sample and 18 patient samples not used for training the classifier. On the non-leukemic sample, only 0.01% of the physiological blood cells were misclassified as being leukemic. These few misclassified cells display physical properties and surface marker expression similar to physiological B-lymphocyte precursors (data not shown). While such immature cells are usually restricted to the bone marrow, they have been described to appear in peripheral blood in small quantities [18]. On the enriched leukemic sample, only 2.88% of the cells were predicted to be non-leukemic cells, a percentage of remaining physiological cells to be expected with the density-gradient centrifugation method for leukemic cell enrichment.

We applied the classifier on separately measured aliquots of one single sample to evaluate the classifier's precision. We observed a maximal difference of 0.8% between the predicted leukemic-cell proportion in any two of these aliquots, underlining the precision of SVM classification on cytometry readouts.

The built SVM classifier was applied to identify leukemic cells in independent patient test samples. In these samples, leukemic cells had been pinpointed beforehand by conventional gating [3]. By SVM-classification, we could recover these leukemic cells with a sensitivity of 99.78% and a specificity of 98.87% (see Table 2). A comparison of the spatial distribution of cells deemed leukemic by manual gating with that of cells classified as leukemic by the SVM shows that both distributions are highly similar, but SVM-predicted leukemic cells encompass a slightly larger area than gated ones (Figure 2).

Nearly all (99.06%) cells were classified congruently by both methods. Importantly, in the day-8 and day-15 samples, taken after the first and second treatment phase, only a small number of incongruently classified cells were observed. The SVM approach successfully recovered the small leukemic cell populations remaining at this stage, thus demonstrating its promising potential in the identification and monitoring of small leukemic subpopulations during leukemia therapy.

Most of the cells that were incongruently classified by manual gating and SVM prediction are deemed non-leukemic by gating but leukemic by the SVM (see Table 2). These cells generally display a light scattering typical of leukemic lymphocytes, and the majority of them show a CD19 and CD34 expression similar to that of leukemic cells detected by manual gating (Figure 3). Their main population is also characterized by a low CD20- and intermediate CD10 expression compatible with a leukemic immunophenotype. However, since their CD10 expression tends to be lower than that of the gating-identified leukemic cells, these cells were not included in the leukemic population by conventional gating.

As such staining variations can arise, e.g., from incomplete staining of cells in the experiment, they decrease the sensitivity of leukemic cell detection by low-dimensional gating. In contrast, the SVM classification is based on all variables at once, and slight variations in only one variable do not hinder the detection of cell populations as long as the remaining variables are characteristic for the sought-after populations. This highlights the strength of the multivariate approach described here.

Also in Figure 3, it can be seen that a small cell subpopulation with low to intermediate CD19 expression was classified as being leukemic by the SVM. Although the CD19 expression of these cells may have been considered below borderline in conventional gating for leukemic B-cells, the artificial noise added to the training data shifted the SVM's decision boundary to include these cells.

The artificial noise seems to be advantageous for learning classification rules in the flow cytometry setting. Various sources of variability arising in the experimental procedure, such as sample contamination, incomplete staining, and instrument instability, can induce shifts in fluorescence and scattering measurements [3]. Classification rules that apply to noisy and noise-free cytometry readouts may be insensitive to such shifts.

Compared to related approaches [4,7], the SVM approach has the advantage that it does not require any control sample group. Thus, it obviates the need to take blood samples from healthy persons. Instead, it is based on a given cell classification, gained from established diagnostic procedures. The SVM approach also does not require a discretization of the numerical data, which would reduce the data's information content, but allows for stable event classification in the high-dimensional space spanned by all measured variables. It does not aim at assigning samples to classes, but rather at assigning single cells to predefined groups. Therefore, no summarization of a variable's distribution over all cells is required.

## Conclusion

The SVM's high classification accuracy is promising, given the fact that the classifier has been build and tested on independent data sets and the training data had been artificially contaminated. Automating the gating for leukemic cells in flow cytometry readouts from blood and bone marrow samples seems highly feasible, even with moderate variations in the experimental procedure.

Furthermore, the SVM automation is applicable to any gating-like procedure for identifying, even small, subgroups of cells in flow cytometry readouts. One of these applications could be the identification of MRD blast cells and monitoring of response to therapy in ALL.

Multivariate classification allows for reliable automation of current diagnostic procedures, taking into account biological dependencies that provide obstacles to simplistic methods. It has the potential to greatly reduce the time, costs and arbitrariness associated with these procedures and allows for shifting efforts to potential research extensions.

In addition, our results show that classification techniques, whose use is already well established on common biological data types, such as gene expression data, can give rise to new algorithms for the analysis of various other existing and upcoming kinds of biological high-throughput data.

## Methods

### Cells and cell staining

Leukemic samples (n = 37) from a series of patients with childhood precursor B-cell ALL (PBC-ALL) collected at initial diagnosis (d0-samples from bone marrow, BM, or peripheral blood, PB), after seven days of initial therapy (d8-samples from PB) and after two weeks of initial therapy (d15 samples from BM) were investigated. In addition to the leukemic samples, an artificial dilution series (n = 6 custom-built mix samples) was generated by mixing blood cells, which had been enriched in leukemic cells by Ficoll density gradient centrifugation, with whole peripheral blood cells from a healthy donor. The sample data on all 43 samples investigated, including estimated percentage of leukemic cells, are shown in Table 1. The cell samples were processed using a BD FACS Lysing Solution (Becton Dickinson, San Jose, CA), according to the manufacturer's instructions, and subsequently stained with a 4-color combination of fluorochrome-conjugated monoclonal antibodies: anti-CD10 phycoerythrin (Dako, Glostrup, Denmark), anti-CD20 fluorescein (Becton Dickinson), anti-CD34 phycoerythrin-cyanin 5.1, and anti-CD19 phycoerythrin-cyanin 7 (Coulter-Immunotech, Hialeah, FL). Measurements of antigen expression were performed by multi-parameter flow cytometry using a FC500 flow cytometer equipped with the Cytomics RXP Analysis Version 1.0 software (Beckman Coulter, Miami, FL). Instrument setup as well as calibration and compensation procedures have been performed according to the recommendations given in [19] and as described in [1]. Analog signals were digitized at 1024-channel resolution. For each sample, the two light-scattering variables FSC and SSC plus four surface markers, CD20, CD10, CD34, and CD 19, were quantified for (50 – 300) 10^3 ^cells. Readouts from the flow cytometry experiments were provided in a standardized file format, called Flow Cytometry Standard (FCS), version 3.0 [20]. The readouts were compensated to reduce effects stemming from overlapping emission spectra of utilized fluorochromes.

### Manual gating

Upon inspection of cells in a two-dimensional dot-plot, cells within a region of interest can be marked, by manually drawing a polygon around them. Many algorithms exist to determine which cells are within the drawn polygon. Once determined, these cells can be highlighted in color, and/or separated from the other cells for further visualization or computations. Currently in leukemia research, cells, which are possibly leukemic, are pinpointed by researchers manually drawing polygons around cells in a number of two-variable dot plots. Shape and position of such defined regions depend on the subjective expertise of the researcher and are not restricted to rectangles at fixed positions. Finally, candidates for leukemic cells are those inside a Boolean combination of drawn regions, called *gate *[2,3].

### Implementation

All computational methods were implemented in the statistical programming language R [21]. Analyses were conducted using custom functions, which depend on released R packages. We used the SVM implementation of the package *e1071 *[22]. Our custom functions have been assembled into a new R-package, called *cytomics*, which is available from the authors upon request.

## Authors' contributions

JT developed the algorithm, performed the computational analysis and wrote the manuscript. PR performed the flow cytometric analysis and contributed to the manuscript. RR provided patient samples and acquired the list mode data. LK designed the project and assisted in writing of the manuscript. RS designed the project, developed the algorithm and assisted in writing of the manuscript. All authors read and approved the final manuscript.

## Figures and Tables

**Table 1:** Sample characteristics. Sample No.: Sample Number, Patient No.: Patient Number (S: custom-built mix samples), Day: Day of treatment (d0: before initial treatment, d8: after first week of treatment, d15: after second week of treatment), Source: Source of sample (BM: bone marrow, PB: peripheral blood, Co: control), Man.% Leukemic: Percentage of events deemed leukemic by manual gating. SVM % Leukemic: Percentage of events deemed leukemic by SVM prediction.

**Sample No.**	**Patient No.**	**Day**	**Source**	**Man. % Leukemic**	**SVM % Leukemic**
1	I	d8	PB	5.88	5.88
2	II	d8	PB	0.04	0.03
3	III	d0	BM	66.33	64.28
4	III	d0	PB	68.09	67.92
5	III	d8	PB	0.26	0.32
6	IV	d0	BM	82.28	82.05
7	IV	d0	PB	6.23	6.12
8	V	d0	BM	31.83	30.82
9	V	d0	PB	13.76	13.17
10	III	d15	BM	0.00	0.03
11	IV	d8	PB	0.68	0.66
12	V	d8	PB	0.08	0.08
13	VI	d0	BM	86.32	88.13
14	VI	d0	PB	56.59	56.97
15	VII	d0	BM	82.41	82.63
16	VII	d0	PB	44.67	44.64
17	VIII	d0	BM	82.87	91.31
18	VIII	d0	PB	59.34	62.34
19	IX	d0	BM	78.59	78.44
20	IX	d0	PB	41.81	41.76
21	X	d0	BM	38.18	37.63
22	X	d0	PB	8.93	9.15
23	IV	d15	BM	0.22	0.50
24	V	d15	BM	0.22	0.27
25	VIII	d8	PB	0.33	0.47
26	VII	d8	PB	0.96	0.97
27	VI	d8	PB	0.46	0.53
28	IX	d8	PB	0.93	1.02
29	XI	d0	BM	29.35	30.05
30	XI	d0	PB	11.24	11.13
31	X	d8	PB	0.81	0.82
32	VIII	d15	BM	0.48	0.40
33	VII	d15	BM	0.55	0.52
34	VI	d15	BM	0.19	0.21
35	IX	d15	BM	1.01	1.02
36	X	d15	BM	0.41	0.49
37	XI	d15	BM	0.02	0.02
38	s	Co		0.00	0.01
39	s	Co		100.00	97.12
40	s	0.01		0.01	0.01
41	s	0.1		0.10	0.08
42	s	1.0		1.00	0.82
43	s	10		10.00	9.41

**Table 2:** SVM prediction on test data. This table displays the numbers of true and wrong predictions by the SVM classifier on test data, taken from 18 patient samples not involved in training the classifier. Rows hold the category the cells have been assigned to by manual gating, columns hold the SVM predicted category. In brackets: Percentage of cells in that gating-assigned category.

	**SVM Leukemic**	**SVM Physiological**	**Total**
**Leukemic by gating**	37,536 *(99.78%)*	83 *(0.22%)*	37,619
**Physiological by gating**	1,602 *(1.13%)*	140,779 *(98.87%)*	142,381
**Total**	39,138 *(27.7%)*	140,862 *(78.3%)*	180,000
